# Supplementary material for: Optimal controller design for reactor core power stabilization in a pressurized water reactor: Applications of gold rush algorithm
Source: PLoS One. 2024 Jan 26;19(1):e0296987. doi: 10.1371/journal.pone.0296987 (PMC10817221; doi:10.1371/journal.pone.0296987)
Supplement: Attachment — Submitted filename: Response to reviewers for PLOS.docx [file pone.0296987.s001.docx]

****Technical response to the reviewers****	October 21^th^, 2023

**Journal name: PLOS ONE**

**Manuscript No.: PONE-D-23-30160**

**Title:** “**Optimal Controller Design for Reactor Core Power Stabilization in a Pressurized Water Reactor: Applications of Gold Rush Algorithm”**

H. Abdelfattah^1^, M. Esmail^1^, Said A. kotb^2^, Mohamed Metwally Mahmoud^3^, Hany S. Hussein4,5, Daniel Eutyche Mbadjoun Wapet^6, *^, Ahmed I. Omar^7^, Ahmed M. Ewais^3^

^1^ Electrical Department, Faculty of Technology and Education, Suez University, 41522, Suez, Egypt

^2^ Reactors Department, Egyptian Atomic Energy Authority (EAEA)

^3^Electrical Engineering Department, Faculty of Energy Engineering, Aswan University, Aswan 81528, Egypt

^4^Electrical Engineering Department, College of Engineering, King Khalid University, Abha 62529, Saudi Arabia

^5^Electrical Engineering Department, Aswan Faculty of Engineering, Aswan University, Aswan 81452, Egypt

^6*^National Advanced School of Engineering, Universit´e de Yaound´e I, Yaound´e, Cameroon

^7^ Electrical Power and Machines Engineering Department, The Higher Institute of Engineering at El-Shorouk City, El-Shorouk Academy, Cairo 11837, Egypt

[hany.abdelfattah@ind.suezuni.edu.eg](mailto:hany.abdelfattah@ind.suezuni.edu.eg), [m.e.hasanin55@gmail.com](mailto:m.e.hasanin55@gmail.com), saidabdou74@yahoo.com,

Metwally_M@aswu.edu.eg, hany.hussein@aswu.edu.eg, [eutychedan@gmail.com](mailto:eutychedan@gmail.com), a.omar@sha.edu.eg

[ewaisa@aswu.edu.eg](mailto:ewaisa@aswu.edu.eg)

*Corresponding author: Daniel Eutyche Mbadjoun Wapet

**Dear Editors and Reviewers**

The authors are thankful to the learned Editor and Reviewers for their thoughtful and detailed comments to improve the quality of the manuscript. The authors have given reviewer comments a lot of interest in the revision process in an attempt to address all of the reviewers’ concerns and corrections as you will already find them incorporated in the revised manuscript. Moreover, a reply to each of the reviewers’ comments is provided below.

Kindly find the response to the reviewer’s comments in the following paragraphs. We hope this revised version of the manuscript meets the editor and reviewers’ expectations, and the standards of publication in the **PLOS ONE Journal.**

The changes carried out by the authors are incorporated in the revised manuscript and highlighted in YELLOW.

**Editor's Comments:**

**Comments to the Authors:**

**Comment-1: *Thank you for submitting your manuscript to PLOS ONE. After careful consideration, we feel that it has merit but does not fully meet PLOS ONE’s publication criteria as it currently stands. Therefore, we invite you to submit a revised version of the manuscript that addresses the points raised during the review process.*** ***The authors must address all the comments of the reviewers and necessary changes must be made in the revised manuscript.***

**Response-1:** Our sincere thanks and appreciation to the editor for considering our manuscript for publication in PLOS ONE Journal, and the recommending submission of the revised manuscript. To improve the quality of the manuscript, the reviewer's queries are addressed and their suggestions are incorporated into the revised manuscript. A new method called hybrid bat algorithm-balloon effect identifier optimizer is designed and implemented and compared with integral controller and Jaya+BE for frequency stability. The introduction section is rewritten, many changes in title, abstract, simulation results, and conclusions are done based on reviewers’ quires. Keywords are rearranged in aphetic order. Some sentences have been edited in the revised paper to clarify the paper's contributions and enhance the paper quality.

**Comment-2:** ***Please include the following items when submitting your revised manuscript:***

***A rebuttal letter that responds to each point raised by the academic editor and reviewer(s). You should upload this letter as a separate file labeled 'Response to Reviewers'.***

***A marked-up copy of your manuscript that highlights changes made to the original version. You should upload this as a separate file labeled 'Revised Manuscript with Track Changes'.***

***An unmarked version of your revised paper without tracked changes. You should upload this as a separate file labeled 'Manuscript'.***

**Response-2:** Our sincere thanks and appreciation to the editor for his comment. The required items are attached during submission process. A cover letter is provided and prepared to explain, point by point, the details of the revisions to the manuscript. The changes carried out by the authors are incorporated in the revised manuscript and highlighted in YELLOW to be easily viewed by the editors and reviewers. An unmarked version of the revised paper without tracked changes is also provided.

**Comment-3: *Please ensure that your manuscript meets*** ***PLOS ONE's style requirements.***

**Response-3:** The authors are extremely thankful to the editor for this thoughtful point. The revised manuscript meets PLOS ONE's style.

**Reviewers Comments:**

**Reviewer 1**

**Comments to the Authors:**

**Comment-1: *This manuscript discusses the evaluation and optimization of the pressurized water reactor (PWR) which is a dynamic model controlled by a PID controller optimized by the gold rush optimizer (GRO). The study concludes that the suggested optimized PID control strategy has superior efficiency and resilience in terms of less overshoot and settling time.***

***In general, the paper is well written. The state of the art is fine, and the included comparisons defending the goodness of the proposal against other existing and recent optimization methods are really good. This paper follows a clear and logical sequence. In addition, the contribution is complete and clear. However, I have a few suggestions to strengthen the paper further:***

***1. I suggest*** ***proofreading the manuscript, as there are several phrases that need improvements, for example: The spelling of optimisation is a non-American variant. For consistency, consider replacing it with the American English spelling (optimization).***

**Response-1:** At the beginning, the authors are thankful to the honorable reviewer for the words of encouragement and trust in our work. As suggested by the esteemed reviewer, proofreading is done in the updated manuscript. The authors apologize regarding these typos. Kindly check the revised manuscript.

**Comment-2: *In section 4, the results and discussion must be accompanied by accurate and complete explanation and justification, for example: Table 1, and figure 7 should be explained and discussed in more detail.***

**Response-2:** The authors are thankful to the esteemed reviewer upon his valuable comment. Based on the reviewer comment, Table 1, and Figure 7 have been explained and discussed in more detail in the updated paper. All the reviewer suggestions are done in the updated paper. Kindly check the revised manuscript.

**Comment-3: *I suggest the authors identify the limitations of their approach.***

**Response-3:** The authors are thankful to the esteemed reviewer upon his valuable comment. The needs and benefits of this study is provided in the updated paper. The proposed method did not appear in any published research article, so it is needed to investigate this method performance.

Despite the good response achieved by using algorithms, the door is opened to more enhancements in system responses during difficulties such as parameters changes. Addition explanation has been added to clarify the need of the proposed algorithm. Kindly check the revised manuscript.

**Comment-4: *I suggest including a future work section, which could provide valuable insights and offer directions for further research.***

**Response-4:** The authors are thankful to the esteemed reviewer upon his valuable comment. Based on the esteemed reviewer suggestion, including a future work section is done in the updated paper. Kindly check the revised manuscript.

**Mai Shawkat add at least 3 references.**

**Reviewer 2**

**Comment-1: *This article is another example of the application of the GRO technique. There are some issues as follows: 1. Why do the authors need to use GRA for tuning the PID controller of the Reactor core power stabilization in a Pressurized Water Reactor? What are the motivations? In the introduction, the authors mention the reference [30] for the inspiration. The authors should explain more about this.***

**Response-1:** At the beginning, the authors are thankful to the honorable reviewer for the words of encouragement and trust in our work. The authors need to use GRA for tuning the PID controller of the reactor core power stabilization in a pressurized water reactor to test this algorithm effectiveness in enhancing the system dynamic performance. The motivations of this study are highlighted in the updated paper. More explanation is provided for the mentioned reference in the updated paper. Kindly check the revised manuscript.

**Comment-2: *The authors claim that the GRA has many benefits over the other methods used in the comparison. Why did the authors select DA, AOA, and PA as comparison methods?***

**Response-2:** The authors are thankful to the esteemed reviewer upon his valuable comment. The authors are thankful to the esteemed reviewer upon his valuable comment. Many industrial applications have employed the DA, AOA, and PA to adaptively adjust the gains of traditional controllers, so these are considered for comparison.

**Comment-3:** ***Why does the system's response in each row shown in Figure 8 look the same?***

**Response-3:** The authors are extremely thankful to the reviewer for this thoughtful point. The system's response in each row shown in Figure 8 look the same is the normal because the changed is the controller setting only. However, in this figure the proposed algorithm proves its accuracy and effectiveness. Kindly check the revised manuscript.

**The authors once again thank the learned Editors and Reviewers for their valuable comments for improving the quality of the manuscript.**
