# Supplementary material for: Optimal controller design for reactor core power stabilization in a pressurized water reactor: Applications of gold rush algorithm
Source: PLoS One. 2024 Jan 26;19(1):e0296987. doi: 10.1371/journal.pone.0296987 (PMC10817221; doi:10.1371/journal.pone.0296987)
Supplement: Attachment — Submitted filename: Response to reviewers for PLOS.docx [file pone.0296987.s002.docx]

****Technical response to the reviewers****	November 24^th^, 2023

**Journal name: PLOS ONE**

**Manuscript No.: PONE-D-23-30160**

**Title:** “**Optimal Controller Design for Reactor Core Power Stabilization in a Pressurized Water Reactor: Applications of Gold Rush Algorithm”**

H. Abdelfattah^1^, M. Esmail^1^, Said A. kotb^2^, Mohamed Metwally Mahmoud^3^, Hany S. Hussein4,5, Daniel Eutyche Mbadjoun Wapet^6, *^, Ahmed I. Omar^7^, Ahmed M. Ewais^3^

^1^ Electrical Department, Faculty of Technology and Education, Suez University, 41522, Suez, Egypt

^2^ Reactors Department, Egyptian Atomic Energy Authority (EAEA)

^3^Electrical Engineering Department, Faculty of Energy Engineering, Aswan University, Aswan 81528, Egypt

^4^Electrical Engineering Department, College of Engineering, King Khalid University, Abha 62529, Saudi Arabia

^5^Electrical Engineering Department, Aswan Faculty of Engineering, Aswan University, Aswan 81452, Egypt

^6*^National Advanced School of Engineering, Universit´e de Yaound´e I, Yaound´e, Cameroon

^7^ Electrical Power and Machines Engineering Department, The Higher Institute of Engineering at El-Shorouk City, El-Shorouk Academy, Cairo 11837, Egypt

[hany.abdelfattah@ind.suezuni.edu.eg](mailto:hany.abdelfattah@ind.suezuni.edu.eg), [m.e.hasanin55@gmail.com](mailto:m.e.hasanin55@gmail.com), saidabdou74@yahoo.com,

Metwally_M@aswu.edu.eg, hany.hussein@aswu.edu.eg, [eutychedan@gmail.com](mailto:eutychedan@gmail.com), a.omar@sha.edu.eg

[ewaisa@aswu.edu.eg](mailto:ewaisa@aswu.edu.eg)

*Corresponding author: Daniel Eutyche Mbadjoun Wapet

**Dear Editors and Reviewers**

The authors are thankful to the learned Editor and Reviewers for their thoughtful and detailed comments to improve the quality of the manuscript. The authors have given reviewer comments a lot of interest in the revision process in an attempt to address all of the reviewers’ concerns and corrections as you will already find them incorporated in the revised manuscript. Moreover, a reply to each of the reviewers’ comments is provided below.

Kindly find the response to the reviewer’s comments in the following paragraphs. We hope this revised version of the manuscript meets the editor and reviewers’ expectations, and the standards of publication in the **PLOS ONE Journal.**

The changes carried out by the authors are incorporated in the revised manuscript and highlighted in YELLOW.

**Editor's Comments:**

**Comments to the Authors:**

**Comment-1: *Thank you for submitting your manuscript to PLOS ONE. After careful consideration, we feel that it has merit but does not fully meet PLOS ONE’s publication criteria as it currently stands. Therefore, we invite you to submit a revised version of the manuscript that addresses the points raised during the review process.*** ***The authors must address all the comments of the reviewers and necessary changes must be made in the revised manuscript.*** ***The reviewers forwarded the comments. The authors must address all the comments of the reviewers and modify the manuscript.***

**Response-1:** Our sincere thanks and appreciation to the editor for considering our manuscript for publication in PLOS ONE Journal, and the recommending submission of the revised manuscript. To improve the quality of the manuscript, the reviewer's queries are addressed and their suggestions are incorporated into the revised manuscript. Some sentences have been edited in the revised paper to clarify the paper's contributions and enhance the paper quality.

**Comment-2:** ***Please include the following items when submitting your revised manuscript:***

***A rebuttal letter that responds to each point raised by the academic editor and reviewer(s). You should upload this letter as a separate file labeled 'Response to Reviewers'.***

***A marked-up copy of your manuscript that highlights changes made to the original version. You should upload this as a separate file labeled 'Revised Manuscript with Track Changes'.***

***An unmarked version of your revised paper without tracked changes. You should upload this as a separate file labeled 'Manuscript'.***

**Response-2:** Our sincere thanks and appreciation to the editor for his comment. The required items are attached during submission process. A cover letter is provided and prepared to explain, point by point, the details of the revisions to the manuscript. The changes carried out by the authors are incorporated in the revised manuscript and highlighted in YELLOW to be easily viewed by the editors and reviewers. An unmarked version of the revised paper without tracked changes is also provided.

**Comment-3: *Please ensure that your manuscript meets*** ***PLOS ONE's style requirements.***

**Response-3:** The authors are extremely thankful to the editor for this thoughtful point. The revised manuscript meets PLOS ONE's style.

**Reviewers Comments:**

**Reviewer 2**

**Comments to the Authors:**

**Comment-2: *I have no more issues with the paper. The paper is well written. The authors have improved the paper according to the reviewer’s comment.***

**Response-1:** At the beginning, the authors are thankful to the honorable reviewer for the words of encouragement and trust in our work.

**Reviewer 3**

**Comment-1: *Appreciating and publishable work but requires few improvements. The paper should be further revised by incorporating the following comments:***

***1. Refine abstract. The abstract should be improved first by explaining a problem and then explaining its proposed solution and benefits over existing strategies.***

**Response-1:** At the beginning, the authors are thankful to the honorable reviewer for the words of encouragement and trust in our work. Based on the esteemed reviewer's suggestion, the abstract is improved in the updated paper. Kindly check the revised manuscript.

**Comment-2: *The introduction should also include the*** ***Research gap and motivation,*** ***Challenges, and Contribution (in points).***

**Response-2:** The authors are thankful to the esteemed reviewer for his valuable comment. The introduction part of the updated paper includes the suggested items. Kindly check the revised manuscript.

**Comment-3:** ***The main contributions of this paper should be further summarized and clearly demonstrated. This reviewer suggests the authors exactly mention what is new compared with existing approaches and why the proposed approach is needed to be used instead of the existing methods.***

**Response-3:** The authors are extremely thankful to the reviewer for this thoughtful point. The authors need to use GRA for tuning the PID controller of the reactor core power stabilization in a pressurized water reactor to test this algorithm effectiveness in enhancing the system dynamic performance. The contributions of this study are highlighted in the updated paper. Kindly check the revised manuscript.

**Comment-4: *PID is not a new controller.***

**Response-4:** The authors are extremely thankful to the reviewer for this thoughtful point. The PID controller is used in industrial till now, and we apply a new optimization. Kindly check the revised manuscript.

**Comment-5: *Objective function (OF) is not new.***

**Response-5:** The authors are extremely thankful to the reviewer for this thoughtful point. The three parameters of the PID controller ($K_{P},K_{I}$,$K_{D})$ are optimized in this work using GRO to boost the NE reactor's performance during load-following operation. In order to gauge how well the suggested optimization method performs, a comparison was provided between DA, AOA, PA, and GRO algorithms.

As shown in Eq. (23), the objective function (OF) is a set of time-domain integral performance indices. The main purpose is to estimate the minimum values of an index function in order to determine the best PID control strategy settings. The integral of time-weighted absolute error (ITAE) motivates the selection of an objective function that seeks to minimize the temporal response characteristics. Furthermore, this OF proves its accuracy and effectiveness in different engineering problems.

$J_{1}=ITAE=\int_{0}^{Ts} t\left| e(t) \right|dt$	(23)

**Comment-6: *All result lines should not be solid******. Differentiate them via solid, dashed, dot, dot-dashed etc.***

**Response-6:** The authors are extremely thankful to the reviewer for this thoughtful point. Based on the esteemed reviewer suggestion, differentiate all result lines via solid, dashed, dot, dot-dashed etc. is performed in the updated paper. Kindly check the revised manuscript.

**Comment-7: *More results should be given.***

**Response-7:** The authors are extremely thankful to the reviewer for this thoughtful point. Two test scenarios are provided to evaluate the suggested controller's efficacy. The first scenario involves constant reference power, whereas the second involves fluctuating reference power. A comparison through four various optimization methods is introduced to judge the presented GRO-tuned PID controller. These four optimizations-PID controllers are DA, AOA, PA, and GRO. In addition, future research directions are provided at the end of this paper. Kindly check the revised manuscript.

**Comment-8: *Results with different nonlinearities should be given.***

**Response-8:** The authors are extremely thankful to the reviewer for this thoughtful point. The second scenario involves fluctuating reference power to show the superiority of the proposed optimized controller. Kindly check the revised manuscript.

**Comment-9:** ***Add corresponding reference to tables/figures.***

**Response-9:** The authors are extremely thankful to the reviewer for this thoughtful point. Adding the corresponding reference to tables/figures is done in the updated paper. Kindly check the revised manuscript.

**Comment-10: *Robustness of the method should also be validated in frequency/time domain.***

**Response-10:** The authors are extremely thankful to the reviewer for this thoughtful point. Two test scenarios are provided to evaluate the suggested controller's efficacy. The first scenario involves constant reference power, whereas the second involves fluctuating reference power. A comparison through four various optimization methods is introduced to judge the presented GRO-tuned PID controller. These four optimizations-PID controllers are DA, AOA, PA, and GRO. In this study, the reactor core of the PWR scheme is utilized simultaneously through the controllers shown utilizing MATLAB/SIMULINK.

**Comment-11: *Compare the results with existing/published results.***

**Response-11:** The authors are extremely thankful to the reviewer for this thoughtful point. Comparing the results with published works is presented in the literature review part. Kindly check the revised manuscript.

**Comment-12: *Literature review should be more strengthen by adding few more papers like***

***https://doi.org/10.1002/2050-7038.12883; https://doi.org/10.1016/j.jestch.2020.12.023;***

***https://doi.org/10.17341/gazimmfd.841751.***

**Response-12:** The authors are extremely thankful to the reviewer for this thoughtful point. The suggested references are helful and strength the literature review part. Kindly check the revised manuscript.

1. G. Sharma, N. Krishnan, Y. Arya, and A. Panwar, “Impact of ultracapacitor and redox flow battery with JAYA optimization for frequency stabilization in linked photovoltaic-thermal system,” Int. Trans. Electr. Energy Syst., vol. 31, no. 5, 2021, doi: 10.1002/2050-7038.12883.
2. I. Eke, M. Saka, H. Gozde, Y. Arya, and M. C. Taplamacioglu, “Heuristic optimization based dynamic weighted state feedback approach for 2DOF PI-controller in automatic voltage regulator,” Eng. Sci. Technol. an Int. J., vol. 24, no. 4, pp. 899–910, 2021, doi: 10.1016/j.jestch.2020.12.023.
3. Z. YENEN YILMAZ, G. BAL, E. ÇELİK, N. OZTURK, U. GÜVENÇ, and Y. ARYA, “Yük frekans kontrolünde kullanılan ikincil denetleyicilerin optimizasyonuna yönelik yeni bir hedef fonksiyonu tasarımı,” Gazi Üniversitesi Mühendislik Mimar. Fakültesi Derg., vol. 36, no. 4, pp. 2053–2068, 2021, doi: 10.17341/gazimmfd.841751.

**The authors once again thank the learned Editors and Reviewers for their valuable comments for improving the quality of the manuscript.**
